# Dynamic regulation of the endocannabinoid system: implications for analgesia

**DOI:** 10.1186/1744-8069-5-59

**Published:** 2009-10-08

**Authors:** Devi Rani Sagar, A Gemma Gaw, Bright N Okine, Stephen G Woodhams, Amy Wong, David A Kendall, Victoria Chapman

**Affiliations:** 1 School of Biomedical Sciences, University of Nottingham, Nottingham NG7 2UH, UK

## Abstract

The analgesic effects of cannabinoids are well documented, but these are often limited by psychoactive side-effects. Recent studies indicate that the endocannabinoid system is dynamic and altered under different pathological conditions, including pain states. Changes in this receptor system include altered expression of receptors, differential synthetic pathways for endocannabinoids are expressed by various cell types, multiple pathways of catabolism and the generation of biologically active metabolites, which may be engaged under different conditions. This review discusses the evidence that pain states alter the endocannabinoid receptor system at key sites involved in pain processing and how these changes may inform the development of cannabinoid-based analgesics.

## Receptor targets for the endocannabinoids and cannabinoids

The *cannabis sativa *plant contains 60 or more bioactive phytocannabinoid compounds including Δ^9^-THC which is the major psychoactive component [1]. A wide variety of synthetic cannabinoids have been produced which interact with cannabinoid receptors, two of which (CB_1 _and CB_2_) have been cloned. Both of these are inhibitory, G_*i *_protein-coupled receptors that reduce the formation of cyclic AMP [2]. CB_1 _receptor activation also inhibits N-, L-, and P/Q-type Ca^2+ ^channels and activates K^+ ^channels and MAP kinases [for review see [3]]. CB_1 _receptors are present pre-synaptically on axons and terminals of neurones, with little or no expression on dendrites or soma [4] and, therefore, are ideally located for the modulation of synaptic activity. Thus, CB_1 _receptor activation inhibits neurotransmitter release and neuronal excitability. CB_2 _receptors couple to similar signal transduction mechanisms to CB_1 _receptors in terms of their actions on adenylyl cyclase and MAP kinases, but do not share the same interactions with ion channels as CB_1 _receptors [for review see [3]].

A third G protein-coupled receptor, GPR55, binds a number of cannabinoid ligands and, therefore, has been proposed to be a member of the cannabinoid receptor family [[5-8], for review see [9]], although the balance of evidence is not supportive of this classification.

TRPV1 receptors are non-selective ion channels whose location in sensory neurons allows them to gate responses to painful stimuli such as high temperature and low pH [for review see [10]]. TRPV1 are activated by the archetypal endocannabinoid anandamide (AEA), albeit at higher concentrations than those which stimulate CB receptors. AEA has recently been shown to excite C-fibres and produce nociceptive behaviour via the activation of TRPV1 [11]. Under inflammatory conditions, such as in the presence of bradykinin or prostaglandins, the sensitivity of TRPV1 to anandamide is increased [12]. Thus, TRPV1 could be considered to be cannabinoid-sensitive ion channel receptor. Other members of the TRP channel family (e.g. TRPA1) also respond to some synthetic cannabinoids (see below). The CB_1_-independent actions of endocannabinoids at other ion channels, including potassium channels and voltage-gated calcium channels have been previously reviewed [13,14]. CB receptor and G protein-independent blockade of the background potassium channels TASK-1 and TASK-3 by AEA has been reported [15], which would be expected to result in depolarisation of sensory nerves and possible functional enhancement. Conversely, Kim et al. [16] reported that AEA inhibited tetrodotoxin-sensitive and tetrodotoxin-resistant sodium channels in primary sensory nerves. Since this effect was unaltered by either CB_1 _or CB_2 _receptor antagonists, or capsazepine, a direct action on these channels may mediate this inhibition. AEA has also been reported to directly inhibit the function of alpha4beta2 nicotinic acetylcholine receptors, independent of CB_1 _receptors [17]. The contribution of these CB_1_-independent actions of the endocannabinoids to their analgesic effects is yet to be fully explored. In this context, however, 5-HT_3 _receptors have been implicated in the CB_1 _receptor-independent analgesic effects of AEA [18].

There is increasing evidence for cannabinoid receptor-independent effects of cannabinoids mediated through the peroxisome proliferator activator receptor (PPAR) family of nuclear receptors [19-23]. Three major isoforms (α, β and γ-) of this ligand-dependent transcription factor have been identified, with their roles in the regulation of lipid metabolism well characterised and studied. Recent studies have demonstrated the involvement of PPAR-α and γ in a variety of additional physiological processes, including inflammation and pain [22,24-28].

The CB_1 _receptor is expressed in neuronal tissue, both centrally and peripherally, as well as in other peripheral organs. CB_1 _receptors are present at lower densities in the heart, lung, testis, ovary, bone marrow, thymus, uterus and immune cells [29]. The CB_1 _receptor is the most abundant G protein-coupled receptor in the brain [30], with particularly high levels of expression in the striatum, cerebellum, basal ganglia, cerebral cortex and hippocampus [30,31]. The widespread distribution of the CB_1 _receptor is consistent with the multiplicity of effects of cannabinoid agonists, including hypomotility, increased food intake, disruption of short term memory consolidation, antinociception, deficits of executive function, anxiety/anxiolysis and psychotropic effects. CB_1 _receptor density is moderate to high in regions involved in pain transmission and modulation, such as dorsal root ganglia (DRG), spinal cord, thalamus, periaqueductal grey (PAG), amygdala and rostroventromedial medulla [32]. The effects of cannabinoid agonists on brain function have been investigated with functional magnetic resonance imaging. Systemic administration of a non-selective CB_1_/CB_2 _agonist increased regional cerebral blood flow, an indirect index of brain activity, in cortical regions, the hippocampus, PAG, nucleus accumbens and striatum [33]. Thus, the brain regions activated by the cannabinoid ligand correspond well to those regions identified by autoradiographic approaches to express moderate to high levels of CB_1 _receptors.

CB_2 _receptors are mainly expressed at high densities in immune tissues, including macrophages, mast cells, and the spleen. Nevertheless, a putative role of the CB_2 _receptor in the nervous system is becoming apparent. Although early studies failed to identify CB_2 _receptors in the central nervous system, recent work has reported the presence of CB_2 _mRNA in the spinal cord of control rats [34] and CB_2 _receptor protein in brain tissue [35,36]. The functional role of CB_2 _receptors in the CNS is unclear. A functional imaging study demonstrated that CB_2 _receptor antagonism did not alter brain activation evoked by systemic administration of a non-selective cannabinoid agonist [33]. These data suggest that CB_2_-mediated cannabinoid-induced changes in brain activity are minor under control conditions.

## Cannabinoid receptor-mediated analgesia

The analgesic effects produced by activation of CB_1 _receptors have been well described and extensively reviewed [for reviews see [37-39]]. Activation of CB_1 _receptors in the spinal cord [40-42] and in the periphery [43] attenuates nociceptive responses of dorsal horn neurones in naïve rats. Supra-spinal CB_1 _receptors, in a number of discrete brain regions, make an important contribution to the antinociceptive effects of cannabinoids in models of acute/tonic pain [44-49]. The broad distribution of CB_1 _receptors in the brain underpins both their therapeutic effects, such as analgesia, as well as their side-effects. To avoid these psychoactive side-effects, the analgesic potential of selective activation of peripheral and spinal CB_1 _receptors has been studied. Anti-nociceptive effects of a CB_1 _receptor agonist were substantially reduced in mice with CB_1 _receptor gene deletion in the peripheral nociceptors [50]. Thus, it appears that CB_1 _receptor agonists which do not cross the blood brain barrier and, thereby, selectively activate peripheral CB_1 _receptors, may provide a promising analgesic strategy. This concept is supported by earlier work demonstrating that hindpaw injection of CB_1 _receptor agonists produces antinociceptive effects in models of inflammatory and chronic pain [42,43,51-55]. Although in most of these studies the effects of cannabinoid agonists were blocked by CB_1 _receptor antagonism, it is important to note that the peripheral anti-hyperalgesic effects of the cannabinoid agonists ACEA and WIN 55,212-2 were mediated via actions at the TRPA1 ion channel expressed by primary afferent fibres [56].

A number of studies have demonstrated analgesic effects of CB_2 _receptor agonists in models of acute and chronic pain [reviewed elsewhere by [57,58]]. Administration of CB_2 _agonists systemically [59-61] or locally into the hindpaw [60,62] attenuates nociceptive responses in naïve rats. CB_2 _receptors are present in the skin and their activation is reported to release endorphins from keratinocytes, acting via μ opioid receptors to produce analgesia [63]. There is little evidence that spinal [64] or supra-spinal [65] CB_2 _receptors modulate nociceptive responses in naive rats, despite the reported expression of supraspinal CB_2 _receptors (see earlier). There is, however, evidence for a novel functional role of CB_2 _receptors in the spinal cord [64,66,67] and thalamus [65] of neuropathic rats. CB_2 _knockout mice exhibit exacerbated neuropathic pain behaviour, including mirror image pain and enhanced microglia and astrocyte activation, suggesting that up-regulation of CB_2 _receptors in the spinal cord in models of neuropathic pain plays an important role in regulating neuropathic pain behaviour [68]. Indeed, chronic treatment with GW405833, a CB_2 _receptor agonist was able to inhibit activation of microglia and astrocytes and attenuate mechanical allodynia in neuropathic rats *in vivo *[69]. Furthermore, the robust inhibitory effects of CB_2 _receptor activation on neuropathic pain behaviour have been shown to be interferon-γ-dependent [70]. Collectively, there is broad base of evidence supporting a major role of spinal CB_2 _receptors in the modulation of neuropathic pain responses. Importantly, CB_2 _receptor selective agonists have been reported to be devoid of CNS-mediated side effects [71].

## Endocannabinoids

At the present time, five endogenous cannabinoid receptor ligands (endocannabinoids) have been described, of which anandamide (*N*-arachidonyl ethanolamine, AEA) was the first to be identified [72]. Since then, 2-arachidonoyl glycerol [2-AG; [73]], noladin ether [74], virodhamine [75] and *N*-arachidonoyl dopamine [NADA; [76]] have been identified. The structurally-related, *N*-acylethanolamines (NAEs) *N*-oleoyl ethanolamine (OEA) and *N*-palmitoyl ethanolamine (PEA) are also widely distributed in the CNS and periphery, but their classification as endocannabinoids is debatable, given their lack of affinity for CB_1 _and CB_2 _receptors. They are, however, PPAR ligands [77,78].

Endocannabinoids are widely believed to be synthesised on demand (i.e. not stored in any cellular compartment awaiting release) and their actions are rapidly terminated by being taken up into cells where they are subject to enzymatic hydrolysis. The anti-nociceptive effects of exogenously administered endocannabinoids have been well described, AEA has anti-nociceptive effects in behavioural models of acute and chronic pain [for review see [37]]. Similarly, 2-AG reduces pain behaviour in the tail-flick [73] and formalin tests [79].

## Endocannabinoid synthesis

**Figure 1:**
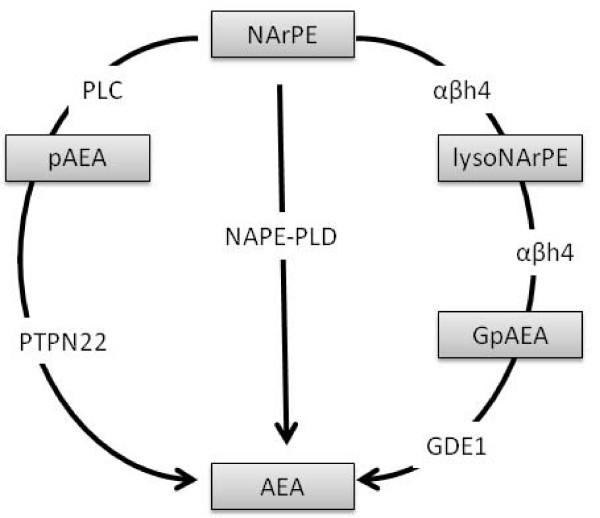
**Proposed biosynthetic pathways for the generation of AEA from its arachidonoyl containing NAPE (NArPE) precursor**. NAPEs are formed from phosphatidyl choline and phosphatidyl ethanolamine membrane precursors by an as yet uncharacterised N-acyl transferase enzyme. The most widely accepted route of AEA biosynthesis is via NAPE-PLD [142,143]. This enzyme is also responsible for the generation of other NAEs including OEA and PEA from their corresponding NAPE precursor. The serine hydrolase αβh4 can generate lysoNAPE and glycerophospho-N-acyl ethanolamine (GpNAE), including glycerophospho-N-arachidonoyl ethanolamine (GpAEA), glycerophospho-N-oleoyl ethanolamine (GpOEA) and glycerophospho-N-palmitoyl ethanolamine (GpPEA) intermediates that are subsequently hydrolysed by a metal dependant phosphodiesterase to produce AEA, OEA and PEA, respectively. In mouse brain, this enzyme has been identified as GDE1 [144]. LPS induced synthesis of AEA involves the generation of phosphorylated AEA (pAEA) via PLC which is then converted to AEA by phosphatases. In mouse brain, this phosphatase has been identified as PTPN22 [83]. Whether this third pathway contributes to the synthesis of other NAEs such as OEA and PEA remains to be determined.

Several different pathways are suggested to contribute to the synthesis of the NAEs, AEA, OEA and PEA from their corresponding *N*-acyl phosphatidyl ethanolamine (NAPE) precursor. The most widely studied pathway to date involves NAPE-phospholipase D (PLD), which generates AEA, OEA or PEA from their precursor, N-arachidonoyl PE (NArPE), N-oleoyl PE or N-palmitoyl PE, respectively (Figure 1) [for review see [80]]. Regionally heterogeneous expression of NAPE-PLD in the mouse brain has been reported [81]. Targeted disruption of NAPE-PLD in mice produces a significant reduction in the brain levels of longer chain NAEs, specifically saturated *N*-acyl chains with 20 or more carbon atoms [82]. By contrast, levels of longer chain polyunsaturated NAEs, including AEA (C20:4) and C22:6 were unaltered in knock-out mice, compared to NAPE-PLD^+/+ ^mice [82]. Thus, NAPE-PLD may not make a substantial contribution to the synthesis of AEA in the brain under control conditions, although this does not preclude an involvement of NAPE-PLD in the synthesis of AEA in discrete brain regions or in the elevated levels of AEA observed following noxious stimulation (see below).

Two alternative pathways involving phospholipase-C (PLC)-PTPN22 [83] and αβ hydrolase (αβH4)-GDE1 [81] are able to generate NAEs, including AEA, OEA and PEA (Figure 1). The functional relevance of these multiple pathways is yet to be determined, but they may subserve differential synthesis of NAEs that might be dependent on the tissue in question. *In vitro *studies have suggested cross-talk between the PLC-PTPN22 pathway and the NAPE-PLD pathway in the generation of AEA [83]. Lipopolysaccharide (LPS) treatment of RAW264.7 cells has been shown to increase levels of AEA, despite reducing NAPE-PLD mRNA. siRNA knockdown of NAPE-PLD in RAW264.7 cells did not alter basal levels of AEA, but increased LPS-stimulated AEA generation, compared to mock-transfected cells [83], providing further support for cross-talk between these synthetic pathways. These *in vitro *data suggest that, in situations where NAPE-PLD generation of AEA is compromised, the PLC-PTPN22 pathway may have a compensatory role in maintaining levels of AEA. Given the lack of effect of the targeted disruption of NAPE-PLD on levels of AEA in the mouse brain, it is feasible that PLC-PTPN22 synthesis of AEA may occur in the NAPE-PLD knockout. The potential role of the PLC-PTPN22 pathway in the synthesis of other NAEs including OEA and PEA remains to be determined.

Recent work has shown that NAPE-PLD independent biosynthesis of NAEs such as AEA, OEA and PEA, occurs via a αβH4-GDE1 pathway in mouse brain and testes [81]. αβH4 is a B-type NAPE lipase capable of removing both *O*-acyl chains from NAPE to yield glycerophosphoNAE (GpNAE) [82]. GDE1, an acyl chain specific phosphodiesterase, then converts GpNAE to NAE [81]. Blockade of this phosphodiesterase activity by EDTA increased levels of long chain polyunsaturated (C20:4, GpAEA; C22:6, GpDHEA) GpNAEs, as well as shorter chain saturated and monounsaturated (C16:0, GpPEA; C18:1, GpOEA) GpNAEs, with no effects on long chain saturated (C20:0) species detected [81]. Further investigation is essential for the understanding of the contribution of these additional synthetic pathways to the maintenance of functional levels of endocannabinoids under control conditions, as well as under different pathological conditions such as chronic pain states which are associated with elevated levels of endocannabinoids.

In contrast to the NAE group of endocannabinoids, the biosynthetic pathways of the acyl glycerols have been less widely studied. Diacylglycerol (DAG), the immediate precursor of 2-AG, is produced from hydrolysis of arachidonate-containing membrane phosphoinositides (PI) or phosphatidic acid (PA) depending on the cell type [for review see; [84]]. Many synthetic pathways for 2-AG upstream of DAG have been proposed in various cell types, which are dependent on phospholipase Cβ (PLCβ) [85]. Two DAG lipases (DAGLα and DAGLβ) catalyse the hydrolysis of DAG to 2-AG [86]. 2-AG synthesis has also been proposed to occur through a phospholipase A1 (PLA1) and phospholipase C (PLC) complementary pathway [87]. DAGLα is located postsynaptically [88] supporting the role of 2-AG as a retrograde messenger [85]. Although DAGL has long been identified and well characterised, its role in modulation of nociceptive processing is only just starting to be clarified. Indeed, DAGLα mRNA is present in the superficial dorsal horn neurones of the spinal cord [89], a region that plays a key role in the processing of nociceptive inputs.

## Endocannabinoids and pain processing

AEA and 2-AG are present in key regions involved in the detection, relay and integration of nociceptive inputs, including the skin, DRG, spinal cord, PAG and rostral ventromedial medulla. Converging evidence supports a role of endocannabinoids in the tonic inhibition of pain responses and the setting of nociceptive thresholds. Indeed, spinal administration of selective CB_1 _receptor antagonists increased evoked-firing of dorsal horn neurones and thermal hyperalgesia [90]. Furthermore, levels of endocannabinoids are altered under pathological conditions such as inflammation and neuropathic pain (Table 1). We have demonstrated a significant reduction in levels of AEA and PEA in the hindpaw of rats with carrageenan-induced hindpaw inflammation [26]. Similarly, levels of AEA, 2-AG and PEA were decreased in the hindpaw following intraplantar injection of formalin [91]. By contrast, Beaulieu *et al*., [92] reported no significant alteration in levels of AEA, 2-AG and PEA in the hindpaw of formalin-treated rats. In addition to altering levels of endocannabinoids at the site of injury, noxious stimulation such as formalin-evoked hindpaw inflammation increases levels of endocannabinoids at other targets in the nociceptive pathway, such as the periaqueductal grey, indicating a role for endocannabinoids in descending control of pain processing [93]. Recent evidence suggests that substance P underlies the 2-AG mediated disinhibition of the descending inhibitory control pathway [94].

**Table 1:** A summary of the changes in levels of endocannabinoid and related compounds in models of inflammatory and neuropathic pain.

***Model***		***Tissue***	***AEA***	***2-AG***	***PEA***	***OEA***	***Reference***
**Inflammatory Pain**							
**Formalin**	Rat	Hindpaw skin	**⇔**	**⇔**	**-**	**-**	[92]

	Rat	Hindpaw skin	**⇔**	**⇓**	**⇔**	**-**	[91]

	Mouse	Hindpaw Skin	**⇓**	**⇓**	**⇓**	**-**	[91]

**Carrageenan**	Rat	Hindpaw skin	**⇓**	**⇓**	**⇓**	**⇔**	[26]

**Neuropathic Pain**							

**Spinal Nerve Ligation**	Rat	L5 DRG	**⇑ (day14)**	**⇑ (day 14)**	**-**	**-**	[95]

	Rat	L4 DRG	**⇔ (day 14)**	**⇔ (day 14)**	**-**	**-**	[95]

	Rat	Lumbar Spinal Cord	**⇑ (day 14)**	**⇑ (day 14)**	**⇓ (day 14)**	**⇔ (day 14)**	[96]

	Rat	Brain (Thalamus)	**⇔ (day 14)**	**⇔ (day 14)**	**⇔ (day 14)**	**⇔ (day 14)**	[65]

**Chronic Constriction Injury**	Rat	Lumbar Spinal Cord	**⇑** **(days 3 & 7)**	**⇑** **(days 3 & 7)**	**⇑ (day 3)** **⇔ (day 7)**	**-**	[139]

	Rat	Brain (PAG)	**⇑** **(days 3 & 7)**	**⇑** **(days 3 & 7)**	**⇔** **(days 3 & 7)**	**-**	[139]

	Rat	Brain (RVM)	**⇔ (day 3)** **⇑ (day 7)**	**⇔ (day 3)** **⇑ (day 7)**	**⇔** **(days 3 & 7)**	**-**	[139]

	Rat	Brain (DR)	**⇔ (day 3)** **⇑ (day 7)**	**⇔** **(days 3 & 7)**	**⇔** **(days 3 & 7)**	**-**	[139]

	Rat	Brain	**⇔ (day 14)**	**⇔ (day 14)**	**-**	**-**	[133]

	Rat	Spinal cord	**⇔ (day 14)**	**⇔ (day 14)**	**-**	**-**	[133]

⇔ = no change, ⇓ = decrease, ⇑ = increase, - = not measured. DRG = dorsal root ganglia, ECB, AEA = anandamide, 2-AG = 2-arachidonyl glycerol, PEA = palmitoyl ethanolamide, OEA = oleoyl ethanolamide, RVM = rostroventral medulla, PAG = periaqueductal gray, DR = dorsal raphe nucleus.

Levels of endocannabinoids and NAEs are altered in different pain states, which may reflect altered synthesis or catabolism. Levels of endocannabinoids are increased in the spinal cord [91] and dorsal root ganglia (DRG) [95] following peripheral nerve injury, a model of neuropathic pain. We have shown that levels of AEA are increased, whereas levels of PEA are decreased, in the spinal cord [96] in a model of neuropathic pain. These data suggest that there is differential synthesis, or catabolism, of AEA and PEA in the spinal cord of neuropathic rats. Neuropathic pain states are associated with activation of glial cells, which contributes to the spinal sensitization and the associated aberrant pain responses [for review see [97]]. As discussed earlier, there is evidence that the biosynthetic pathways responsible for EC synthesis are cell type-dependant. Activated microglia synthesize and metabolize endocannabinoids [98-100] and, therefore, their presence in the spinal cord in models of neuropathic pain is likely to influence the local availability of endocannabinoids under these conditions.

## Endocannabinoid metabolism

**Figure 2:**
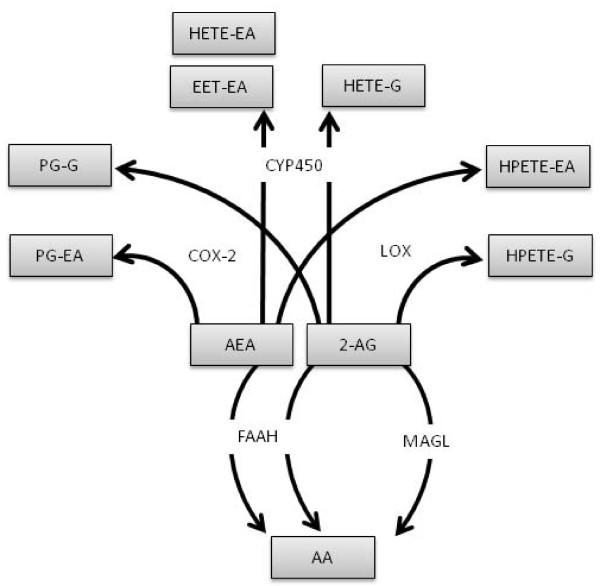
**Proposed metabolic pathways for the breakdown of AEA and 2-AG via hydrolase and oxygenase pathways**.

To date, hydrolase and oxygenase pathways have been shown to be the major pathways responsible for the metabolism of the endocannabinoids, in particular AEA and 2-AG (Figure 2). Hydrolysing enzymes include fatty acid amide hydrolase (FAAH), monoacylglycerol lipase (MAGL) and *N*-acylethanolamine-hydrolysing acid amidase (NAAA). AEA and other NAEs are mainly hydrolysed by FAAH through the hydrolytic cleavage of the amide bond to form arachidonic acid and ethanolamine [101-103]. An additional isoform of FAAH (FAAH2), has been identified which has a limited species distribution in mammals, being found in man and other primates, but not in rodents [104]. FAAH2 appears to be poorly expressed, if at all, in the brain. 2-AG is mainly metabolised by MAGL to arachidonic acid and glycerol [105,106]. NAAA is a lysosomal enzyme with optimum activity at an acid pH. It can metabolise AEA and PEA to their corresponding fatty acids and ethanolamine, but 2-AG is a poor substrate [107]. Levels of NAAA are low in the brain and the enzyme is unlikely to be an important mediator of endocannabinoid metabolism under normal conditions.

AEA and 2-AG are also substrates for the oxidative enzymes cyclooxygenase type-2 [COX-2; for review see; [108]], lipooxygenase (LOX) and cytochrome p450s (CYP450s). Whilst the effects of COX, LOX and CYP450 are not specific to endocannabinoid catabolism, their effects on endocannabinoids are of interest, not least because of the potential for oxidation of the arachidonic acid moiety to generate pharmacologically active metabolites. The proposed biological actions of some of these metabolites are summarised in Table 2. COX-2 is constitutively expressed in the kidney, spinal cord, hippocampus, cortex and hypothalamus [102] and it is up-regulated in pathological states, including inflammatory pain [109]. AEA and 2-AG can be converted by COX-2 to prostamides (prostaglandin-ethanolamides) and prostaglandin glyceryl esters, respectively [[110], for reviews see; [111,112]]. The prostamides are weakly active at cannabinoid CB_1 _and CB_2 _receptors and prostamide F_2α _is a weak agonist at TRPV1 [108,113]. Prostamides D_2 _and E_2 _are present in mouse lung and kidney, with higher levels seen in AEA-treated FAAH knockout mice, compared to control mice. Prostamide F_2α _was only detected in the liver, kidney, lung and small intestine of AEA-treated FAAH knockout mice. [114].

**Table 2:** Summary of known biological actions of the endocannabinoid metabolites, and their effects in models of pain.

***Synonym***	***CB*_1_**	***CB*_2_**	***TRPV1***	***PPAR-α***	***Effects in pain models***	***Reference***
**PGD2-EA**	weak agonist	weak agonist			yet to be shown	[108,113]

**PGE2-EA**		weak agonist			yet to be shown	[108,113]

**PGF2α-EA**	agonist		weak agonist		pro-inflammatory, produces allodynia	[108,113,140,141]

**PGE2-G**					pro-inflammatory, produces mechanical allodynia and thermal hyperalgesia	[108,113,117]

**5,6-EET-EA**		agonist			yet to be shown	[118]

**15-HPETE-G**				agonist	yet to be shown	[102]

**2-(11,12)EG**	agonist	agonist			yet to be shown	[119]

**2-(14,15)EG**	agonist	agonist			yet to be shown	[119]

**2-(14,15)DHETE-G**				agonist	yet to be shown	[121]

PGD2-EA = prostamide D2, PGE2-EA = prostamide E2, PGF2α-EA = prostamide F2α, PGE2-G = prostaglandin E2-glyceryl ester, 5,6-EET-EA = 5,6-epoxyeicosatrienoic acid ethanolamide, 15-HPETE-G = 15-hydroperoxyeicosatetraenoic acid glycerol ester, 2-(11,12)EG = 2(11,12-epoxyeicosatrienoyl)glycerol, 2-(14,15)EG = 2(14,15-epoxyeicosatrienoyl)glycerol, 2-(14,15)DHETE-G = 2-(14,15-dihydroxyeicosatetraenoic acid)-glycerol ester.

Biological effects of the COX2 metabolite of 2-AG, prostaglandin glyceryl esters (PG-Gs) have been demonstrated in the hippocampus, where they modulate GABAergic mediated inhibitory synaptic transmission [115] and enhance hippocampal glutamatergic transmission and neurotoxocity [116]. 2-AG suppresses the elevation of COX2 in response to pro-inflammatory stimuli, thus limiting the generation of neurotoxic products of 2-AG [116]. The potential roles of COX2 metabolites of 2-AG in pain processing have not been widely studied. Intraplantar injection of PGE_2_-glyceryl ester (PGE_2-_G) produced mechanical allodynia and thermal hyperalgesia, suggesting that pro-nociceptive ligands could be generated by the COX2 metabolism of 2-AG *in vivo *[117]. PGE_2_-G is present in the rat hindpaw, but it was below detection limits in the spinal cord and brain in naïve rats and endogenous levels in the hindpaw were unaltered in a model of inflammatory pain [117]. Further studies are required to determine whether PGE_2_-G modulates spinal and/or supraspinal nociceptive processing in models of chronic pain.

The endocannabinoids are also metabolised by LOX and CYP450s. The main isoforms of LOX that metabolise AEA and 2-AG are 5-LOX, 12-LOX and 15-LOX, all of which give rise to different subsets of metabolites. CYP450 enzymes 2D6, 3A4 and 4F2 produce several metabolites of NAEs including 5,6-epoxyeicosatrienoic acid ethanolamide (5,6-EET-EA), which is more stable than AEA in brain homogenate and is a potent and selective CB_2 _agonist *in vitro *[118]. It is of particular interest, in the context of chronic pain states, that activated BV-2 microglial cells have an increased capacity to convert AEA to 5,6-EET-EA, which may have relevance to neuropathic pain states. Indeed, neuropathic pain states are, as discussed earlier, associated with activated microglia, increased levels of AEA in the spinal cord and the novel functional expression of CB_2 _receptors, activation of which attenuates nociceptive responses. The role of metabolites such as 5,6-EET-EA in the modulation of central sensitization in models of chronic pain is unknown, and warrants investigation.

A novel group of CYP450 metabolites of AA has been identified in the spleen, kidney and brain and were termed 2-epoxyeicosatrienoyl-glycerols (2-EGs). Some of these products, 2-(11,2-epoxyeicosatrienoyl)glycerol (2-11,12-EG) and 2-(14,15- epoxyeicosatrienoyl)glycerol (2-14,15-EG) have high affinity for CB_1 _and CB_2 _receptors in transfected CHO cells [119]. 2-EG is present in the brain and systemic administration of 2-EG decreased spontaneous locomotor activity and core body temperature in mice, an effect which was sensitive to CB_1_, but not CB_2 _receptor, blockade. [119]. Whether 2-EG also produces CB_1 _receptor-mediated analgesia remains to be determined.

As well as activating cannabinoid receptors, oxidative metabolites of endocannabinoids also activate the PPAR nuclear receptor family. The anti-inflammatory and analgesic effects of PPARα ligands are discussed below. Both the predominant product formed following incubation of 2-AG with 15-LOX, 15-HPETE-G, and the CYP450 metabolite of arachidonic acid, 8(S)-HETE, [120] are agonists at the PPARα [102]. In addition, 2-(14,15)-DHETE-G, a CYP450 metabolite of 2-AG, produces a four-fold increase in PPAR-α activation in transfected COS-7 cells, suggesting it is also a PPAR-α agonist [121]. It should be kept in mind that there are many biological activators of PPARs and, therefore, it is important to determine that potential agonists can reach intracellular concentrations able to activate these receptors before they are considered to be functionally relevant.

**Figure 3:**
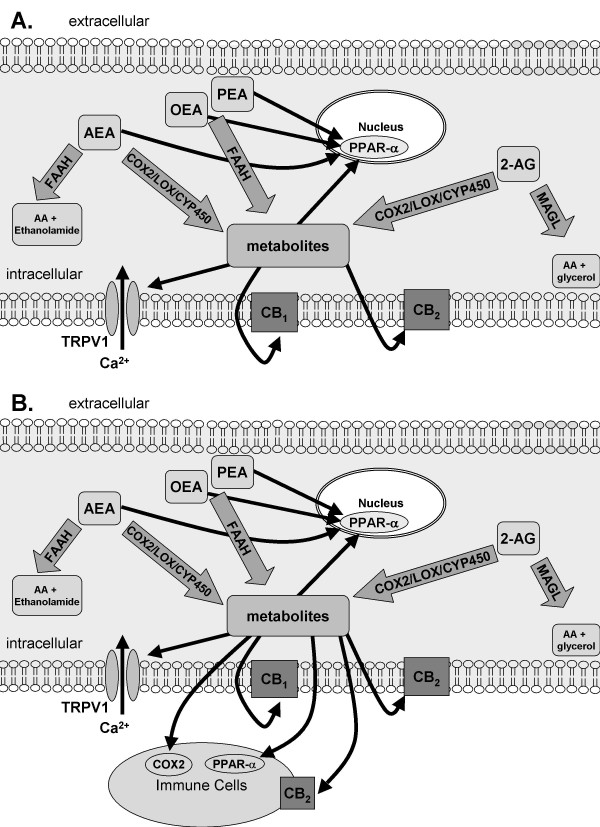
**A: The endocannabinoid AEA and related NAEs PEA and OEA are broken down by FAAH, 2-AG is primarily metabolized by MAGL**. AEA is a ligand at CB_1_, CB_2 _and TRPV1 receptors and the nuclear receptor PPAR-α. OEA and PEA are ligands for PPAR-α. 2-AG is a ligand at CB_1_and CB_2_. Both AEA and 2-AG can be metabolized by COX2, LOX and CYP450 to form biologically active metabolites, some of which are ligands for CB_1_, CB_2 _and PPAR-α. B: Under pathological conditions, such as inflammatory or neuropathic pain, the presence of infiltrating immune cells or the activation of microglia provides another source of endocannabinoid synthesis and catabolism, as well as providing additional/or alternative receptor sites of action of the endocannabinoids, NAEs and their metabolites.

Thus, is is evident that in addition to the more conventional components of the endocannabinoid system, the metabolism of endocannabinoids via the hydrolase and oxidative pathways has the potential to generate various modulators of physiological/pathophysiological processing (Table 2), the generation of which is dependent on the cell types present, their state of activation and the enzymes expressed by these cells (Figure 3). In some cases ligands which act via alternative receptor mechanisms are generated from the endocannabinoids, in other cases more stable ligands for the cannabinoid receptors are generated. Further investigation of the biological significance of these complex metabolic pathways in models of chronic pain states are required to determine whether there are important additional novel analgesic targets that can be exploited.

## Attenuation of endocannabinoid catabolism produces analgesia

Following the extensive study of the analgesic effects of CB_1 _receptor activation in models of acute and chronic pain it was clear that a more selective approach was required to achieve analgesia in the absence of the side-effects produced by global stimulation of cannabinoid receptors. The obvious strategy of preserving endocannabinoids by means of catabolic enzyme inhibition has been employed by a number of research groups. This has the potential to activate simultaneously a variety of relevant targets by a whole range of different endocannabinoids and related compounds that are substrates for the enzyme in question. This has the advantage of promoting endocannabinoid signalling predominantly at those sites at which the neuronal activity is greatest, thereby selectively controlling pain pathways when noxious stimuli are present.

The role of FAAH in the metabolism of endocannabinoids has been demonstrated in mice lacking FAAH (FAAH^-/-^), which exhibit 15 fold elevated levels of AEA, compared to wild-type mice. FAAH^-/- ^mice display phenotypic hypoalgesia in models of acute and inflammatory pain [122,123], but not neuropathic pain [123]. Pharmacological inhibition of FAAH is antinociceptive in models of acute and inflammatory pain [124-129]. A single systemic injection of the FAAH inhibitor URB597 significantly reduced thermal allodynia and mechanical hyperalgesia in the complete Freund's adjuvant (CFA) model of inflammation [124]. In the carrageenan model of inflammation, we reported that intraplantar injection of URB597 increased levels of AEA and 2AG in hindpaw skin and reduced carrageenan-hyperalgesia [26]. Whilst the analgesic effects of these compounds have been clearly demonstrated, the selectivity and efficacy of URB597 has recently been questioned. URB597 is an irreversible FAAH inhibitor which also displays inhibitory activity at multiple additional members of the serine hydrolase family [130]. Reversible inhibitors such as OL-135 display greater selectivity for FAAH, but have reduced efficacy [126]. In light of this, novel FAAH inhibitors such as JNJ1661010 [131] and PF-3845 [132] which have enhanced selectivity and potency have been developed. Both display robust anti-hyperalgesic properties in rat models of inflammatory pain, which are sensitive to blockade of CB_1 _and CB_2 _receptors by SR141716 or SR144528 respectively [131,132].

The effects of inhibition of FAAH on neuropathic pain behaviour are less consistent than those reported for inflammatory pain states. Acute systemic injection of URB597 (0.3 mg/kg, i.p.) did not alter mechanical allodynia in a model of peripheral neuropathy [124]. Similarly, a single oral dose of URB597 (10 mg/kg, p.o.) had limited effects on mechanical hyperalgesia in the chronic constriction injury (CCI) model of peripheral neuropathy [129]. By contrast, repeated administration of URB597 (10 mg/kg, for 4 days p.o.) significantly reduced thermal and mechanical hyperalgesia [129] whilst OL135 (ED50 9 mg/kg i.p.) reduced mechanical allodynia [125] in neuropathic rodents. Inhibition of FAAH by either URB597 or OL135 also reduced mechanical and cold allodynia in CCI mice. These inhibitory effects were blocked by CB_1 _but not CB_2 _or TRPV1 antagonists and were accompanied by raised levels of AEA in the brain and spinal cord [133]. In addition to these studies, repeated subcutaneous administration of URB597, OL-135 (3 mg/kg, 7 days), or AA-5-HT (5 mg/kg, 7 days) from post-operative day 1 ablated the development of mechanical allodynia and thermal hyperalgesia in the rat CCI model of neuropathic pain [91]. Collectively, these data suggest that there is an alteration in synthesis/metabolism of endocannabinoids and endocannabinoid-like compounds, or their receptor function, in models of neuropathic pain, which is supported by data from our electrophysiological studies [134].

The focus of research in this area has centred on the prevention of AEA catabolism by FAAH, largely due to the paucity of selective inhibitors for the major 2-AG catabolic enzyme; MAGL [79,102,135]. Recently a novel compound, JZL184, which has >300 fold selectivity for MAGL over FAAH *in vitro*, has been described [135]. JZL184 significantly increases levels of 2-AG *in vivo *and produces analgesia in mouse models of acute and inflammatory pain [136]. JZL184 also attenuated mechanical and cold allodynia in CCI mice, effects which were mediated by the CB_1 _receptor and were accompanied by raised levels of 2-AG in the brain and spinal cord [133]. Further use of this and other recently described compounds (e.g. OMDM169 [137]) alone and in conjunction with existing inhibitors will provide greater insight into the respective roles of 2-AG and AEA in pain states, and aid the future development of analgesics based on attenuation of EC catabolism.

## Role of PPARs in mediating analgesic effects of FAAH inhibition

There is increasing evidence that, in addition to cannabinoid receptor mediated analgesia, NAEs such as PEA produce analgesia via activation of nuclear receptors (Figure 3). PEA is an endogenous ligand of PPAR-α and peripheral administration of PEA rapidly reduces formalin-evoked nocifensive behaviours and neuronal activity in mice [77]. The role of the PPAR-α in the analgesic effects of PEA was confirmed by the absence of these effects in PPAR-α null mice. Although PPAR-α is a nuclear receptor, the rapid onset of these effects suggests that mechanisms independent of gene transcription, which may include central sites of action, may contribute to these effects, [138]. In addition to its effects at PPAR-α, anti-allodynic and antihyperalgesic effects of PEA are mediated, at least in part, by PPAR-gamma [19]. We have demonstrated that the inhibition of inflammatory pain behaviour associated with the increase in levels of AEA and NAEs, produced by inhibition of FAAH and COX-2, is mediated at least in part through activation of PPAR-α [26]. Electrophysiological studies in our group have confirmed the role of PPAR-α in mediating the effects of URB597 on carrageenan-evoked receptive field expansion [27]. Although further studies are required, it is likely that the contribution of PPAR-α in mediating the effects of FAAH and COX2 inhibition arises as a result of the presence of additional targets in inflammatory pain states, for example infiltrating immune cells (Figure 3).

In conclusion, cannabinoid ligands produce well documented analgesic effects mediated by the CB_1 _and CB_2 _receptors; however, other receptor systems may also contribute, in particular in inflammatory and neuropathic pain states (Figure 3). The emerging evidence that the levels of cannabinoid receptors, their ligands and biologically active metabolites are altered in a tissue-specific manner under pathological conditions, such as chronic pain states, may support a more targeted approach to the development of cannabinoid-based analgesics.

## Abbreviations

2-AG: 2-arachidonoyl glycerol; 2-EGs: 2-epoxyeicosatrienoyl-glycerols; AA-5HT: N-arachidonoyl serotonin; AEA: N-arachidonoyl ethanolaminem, Anandamide; cAMP: cyclic adenosine monophosphate; CB_1_: Cannabinoid 1 receptor; CB_2_: Cannabinoid 2 receptor; CCI: Chronic constriction injury; CFA: Complete Freunds adjuvant; COX-2: Cylcooxygenase type 2; CYP450: Cytochrome P450; DAG(L): Diacylglycerol (Lipase); DHETE-G: Dihydroxyeicosatrienoic acid glycerol ester; DR: Dorsal Raphe Nucleus; DRG: Dorsal root ganglion; EET-EA: Epoxyeicosatrienoic acid ethanolamide; FAAH: Fatty acid amide hydrolase; GpAEA: glycerophospho-N-arachidonoyl ethanolamine; GpOEA: glycerophospho-N-oleoyl ethanolamine; GpPEA: glycerophospho-N-palmitoyl ethanolamine; GpNAE - glycerophospho-N-acyl ethanolamine; HETE: Hydroxyeicosatetraenoic acid; HPETE-G: Hydroxyperoxyeicosa-5,8,10,14-tetraenoic acid glycerol ester; i.p.: Intraperitoneal administration; i.pl.: Intraplantar administration; LOX: Lipoxygenase; LPS: Lipopolysaccharide; MAGL: Monoacylglycerol lipase; MAPK: Mitogen activated protein kinase; NAAA: N-acylethanolamine hydrolysing acid amidase; NADA: N-arachidonoyl dopamine; NAE: N-acylethanolamines; NAPE: N-phosphatidyl ethanolamine; NArPE: arachidonoyl containing NAPE; NMDA: N-methyl-D-aspartic acid; OEA: N-oleoyl ethanolamine; pAEA: phosphorylated anandamide; PAG: Periaqueductal grey; PEA: N-palmitoyl ethanolamine; PLA1: Phospholipase A1; PLC: Phospholipase C; PLD: Phospholipase D; p.o.: Oral administration; PPAR: Peroxisome proliferator-activated receptor; PGE_2_-G: Prostaglandin E_2_-glycerol; RVM: Rostrovental Medulla; SNL: Spinal nerve ligation; Δ^9^-THC: Δ^9^-Tetrahydrocannabinol; TRPV1: Transient receptor potential vanilloid type 1

## Competing interests

The authors declare that they have no competing interests.

## Authors' contributions

DRS, AGG, BNO, SGW, AW, DAK and VC contributed to the researching and writing of this manuscript. All authors read and approved the final manuscript.
